# Mortality and Morbidity during Extreme Heat Events and Prevalence of Outdoor Work: An Analysis of Community-Level Data from Los Angeles County, California

**DOI:** 10.3390/ijerph15040580

**Published:** 2018-03-23

**Authors:** Kevin Riley, Holly Wilhalme, Linda Delp, David P. Eisenman

**Affiliations:** 1 UCLA Labor Occupational Safety and Health Program, Los Angeles, CA 90095, USA; ldelp@ucla.edu; 2 Department of Medicine Statistics Core, David Geffen School of Medicine at UCLA, Los Angeles, CA 90024, USA; leclairholly@gmail.com; 3 Division of General Internal Medicine and Health Services Research, David Geffen School of Medicine, University of California at Los Angeles, Los Angeles, CA 90024, USA; deisenman@mednet.ucla.edu; 4 Center for Public Health and Disasters, Fielding School of Public Health, University of California at Los Angeles, Los Angeles, CA 90024, USA

**Keywords:** extreme heat, community health, occupational health, outdoor work, climate change

## Abstract

Heat is a well-recognized hazard for workers in many outdoor settings, yet few investigations have compared the prevalence of outdoor work at the community level and rates of heat-related mortality and morbidity. This analysis examines whether heat-related health outcomes occur more frequently in communities with higher proportions of residents working in construction, agriculture, and other outdoor industries. Using 2005–2010 data from Los Angeles County, California, we analyze associations between community-level rates of deaths, emergency department (ED) visits, and hospitalizations during summer heat events and the prevalence of outdoor work. We find generally higher rates of heat-related ED visits and hospitalizations during summer heat events in communities with more residents working outdoors. Specifically, each percentage increase in residents working in construction resulted in an 8.1 percent increase in heat-related ED visits and a 7.9 percent increase in heat-related hospitalizations, while each percentage increase in residents working in agriculture and related sectors resulted in a 10.9 percent increase in heat-related ED visits. The findings suggest that outdoor work may significantly influence the overall burden of heat-related morbidity at the community level. Public health professionals and healthcare providers should recognize work and employment as significant heat risk factors when preparing for and responding to extreme heat events.

## 1. Introduction

Heat is the primary weather-related cause of death in the United States. The estimated mean annual heat-related death toll in the U.S. is about 1500 [1], with extreme heat events accounting for more fatalities annually than the 30-year mean annual number of deaths due to hurricanes, lightning, earthquakes, tornadoes, and floods combined [2,3]. Extreme heat events also lead to increased rates of non-fatal medical conditions [4,5,6]. Semenza and colleagues, for example, estimated that the July 1995 heatwave in Chicago was responsible for over 1000 excess hospital admissions [7]. Similarly, a study of emergency medical services in Phoenix between 2001 and 2006 concluded that the greatest incidence of heat-related medical dispatches occurred during the times of peak solar irradiance, maximum diurnal temperature, and elevated heat indices (combined temperature and relative humidity) [8]. The frequency and severity of extreme heat events—and the resulting health consequences—are only expected to increase in the coming decades as a result of global climate change [9,10,11,12,13,14].

Among the populations at an elevated risk for environmental heat exposure are individuals working in outdoor settings. Construction and agriculture workers in particular have been shown to experience a greater incidence of heat-related illness and death compared to workers in other industries [15,16,17,18,19]. Prolonged exposure to high temperatures, humidity, and direct sunlight combined with the effects of physical labor render many outdoor workers vulnerable [15,16]. Use of protective clothing and equipment may further exacerbate workers’ risk by preventing the body from cooling, while personal factors such as hydration, medication use, pregnancy, and overall health status also contribute to heat-related health outcomes [20,21]. Frequent and extreme variations in outdoor weather conditions may also complicate workers’ ability to acclimatize [20,21,22]. Policies and regulations can serve to mitigate workers’ exposures to heat-related hazards; however, currently California and Washington are the only states in the U.S. with occupational health standards to prevent heat illness in outdoor work settings [23].

While studies have documented heat-related risk factors and health outcomes that outdoor workers face [20,21,24,25,26], few investigations have considered whether the prevalence of outdoor work contributes to patterns of heat-related mortality and morbidity at the community level. That is, do communities with greater numbers of residents working in construction, agriculture, and other outdoor settings see higher rates of heat-related illness and death? Such an approach departs from the traditional occupational health focus on workplaces and worker populations, instead highlighting work and employment as important characteristics of communities, in which common work activities and work environments serve as social determinants of health that, in turn, contribute to wider community health disparities [27,28,29,30,31]. Health outcomes related to environmental heat exposure offer a unique opportunity to examine the potential relationship between work and health outcomes measured at the community level, since meteorological data allow for easy identification of heat event days; the symptoms associated with heat exposure are often acute in nature and explicitly attributed to heat in coroners’ reports and hospital records; and population-level data on the categories of occupation and industry are widely available through U.S. Census surveys.

Using various data sources for Los Angeles County, California, between 2005 and 2010, we ask: Do the rates of heat-related mortality and morbidity increase at the community level along with the proportion of residents in those communities who work in outdoor industries?

Evidence of associations between outdoor work and heat-related health outcomes can indicate communities in potential need of interventions—both workplace- and community-based—to protect workers from occupational heat exposure. The findings may also offer valuable lessons for planning and surveillance activities related to extreme heat events. Public health researchers and emergency medical service providers have made important advances in identifying community-level risk factors for heat-related mortality and morbidity—from mean population age, to availability of air conditioning and cooling facilities, to proportion of residents living alone [3,32,33,34,35,36]; however, recognition of work and employment as contributing factors has been limited. Emergency alert systems such as the National Weather Service’s Heat Index warnings do not acknowledge the effects of metabolic heat and exertion in direct sunlight, which account for much of the heat-related risks that outdoor workers face and can increase heat index values by as much as 15 degrees Fahrenheit (http://www.nws.noaa.gov/om/heat/heat_index.shtml). CDC programs such as the National Environmental Public Health Tracking Network fail to include work-related risk factors among its indicators of heat vulnerability [36,37]. Additionally, while syndromic surveillance guidelines on climate change and health from the Council of State and Territorial Epidemiologists include recommendations for tracking work-related conditions, their dependence on the accurate recognition and recording of work relatedness by healthcare providers may ultimately limit the usefulness of this approach for population-level surveillance [38].

Our analysis is distinctive in another notable way: Although studies have examined heat-health relationships among outdoor workers based upon temperature and humidity, our research is the first to our knowledge to assess the occupational heat-health relationship using a spatial synoptic classification scheme to characterize entire weather situations [39,40]. This approach incorporates numerous meteorological elements that comprise an air mass, including temperature, humidity, pressure, and wind, along with visibility, cloud cover, and, sometimes, precipitation [41]. This is notable, as outdoor workers are exposed to the simultaneous effects of the numerous meteorological elements that comprise an air mass. The synoptic procedure permits an evaluation of synergistic relations among weather elements; the combined impact of several elements is more significant than the sum of their individual parts. An analysis that identifies those meteorological situations, those air masses, that are most debilitating to human health renders this evaluation unique [42]. Air-mass based approaches are the foundation of “heat-health warning systems” used by a number of National Oceanic and Atmospheric Administration (NOAA)/National Weather Service Offices around the world to call excessive heat warnings [43,44,45,46,47]. Thus, the air mass-based approach is well-suited for use in analyses relevant to both occupational and community health.

## 2. Materials and Methods

The main health outcomes for this analysis were derived from data from the California Office of State Health Planning and the California Department of Public Health Vital Statistics for Los Angeles County, California, between 2005 and 2010. Specifically, we considered deaths, emergency department (ED) visits, and hospital discharges—both those that were identified as heat related and those attributed to All Internal Causes.

Heat-related mortality was defined as any death that had code X30 (exposure to excessive natural heat) or T67 (effects of heat and light) listed as either the underlying cause of death or within the first 20 other causes of death on the death certificate. For ED visits and hospitalizations, heat-related illness was defined as those records with heat-related illness (ICD-9 code 992) or effects of heat and light (ecode E9000) listed in any of up to 20 diagnoses. All Internal Causes of death were those who died of heat-related causes or any of the following causes of death listed as either the underlying cause of death or other causes of death: cardiovascular (I00–I99); respiratory (J00–J99); acute kidney failure and chronic kidney disease (N17–N19); disorders of fluid, electrolyte, and acid-base balance (E87); dehydration (E86); and diabetes (E08–E13). ED visits and hospitalizations attributed to All Internal Causes were those with the following ICD-9 codes listed as a diagnosis: electrolyte imbalance (276); cardiovascular disease (390–398, 402, 404–429, 440–448); cerebrovascular disease (Codes 430–438); respiratory illness (Codes 460–519); nephritis and nephrotic syndrome (Codes 580–589); acute renal failure (Code 584); heat-related illness (Code 992); effects of heat and light (E9000); and diabetes mellitus (Code 250). These various diagnoses have been shown to increase during heat waves [33,48,49].

In order to identify cases of heat-related mortality and morbidity resulting from environmental causes, we confined our analysis to health outcomes (both heat-related and All Internal Cause) occurring on heat event days during summer months (May–September) for each calendar year. Heat event days were identified using spatial synoptic classification of the National Oceanic and Atmospheric Administration’s National Environmental Satellite, Data, and Information Service (2012). The spatial synoptic classification (SSC) system categorizes each day into one of a number of air mass types; we focused on two “oppressive” air masses (and variations thereof) that are associated with statistically significantly higher daily mortality: Dry Tropical (3), Dry Tropical + (33), Moist Tropical (6), Moist Tropical + (66), and Moist Tropical ++ (67) (see http://publichealth.med.miami.edu/ synoptic-climatology-laboratory/synoptic-lab-research). This air mass-based approach was developed by Sheridan [43] and has since been applied in studies relating to climate and human health [46,47]. We used SSC data from Burbank Airport to determine summer heat event days. (Los Angeles features a wide variety of microclimates—from tropical coastal zones to high mountains and dry desert regions. Burbank Airport served as a more “representative” measure of countywide air mass conditions than Los Angeles International Airport, given the latter facility’s proximity to the coast.) For purposes of this analysis of occupational heat exposure, we also limited our focus to mortality and morbidity among adults aged 16 and over.

We examined rates of deaths, ED visits, and hospitalizations at the zip code level in Los Angeles County and considered them in relation to the proportion of residents living in those zip codes who are employed in outdoor industries. Five-year estimates from the U.S. Census Bureau’s 2012 American Community Survey were used to extract zip code level measures of the proportion of residents working in either of two industry categories: “construction” or “agriculture, forestry, fishing and hunting, and mining.” For comparison purposes, we also considered associations between our select health outcomes and prevalence of work in the “non-outdoor” sectors of “education services, and healthcare and social assistance.” (The American Community Survey only reports industry data using the broadest North American Industry Classification System (NAICS) categories, and, in some cases, ACS aggregates industry data across categories. Agriculture, forestry, fishing and hunting (NAICS code 11), for example, is combined with mining (NAICS code 21), while education services (NAICS code 61) is combined with healthcare and social assistance (NAICS code 62). As a result, we were unable to isolate certain other outdoor industries—landscaping, select transportation sectors, etc.—for analysis. While there is little mining activity in Los Angeles County, oil and gas drilling is included under this broad NAICS category and is common within the county).

**Figure 1:**
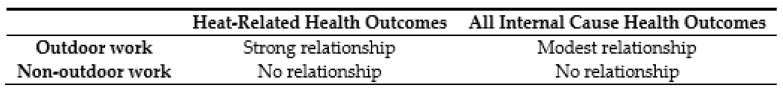
Anticipated relationships between variables of interest.

Figure 1 summarizes the relationships we expected to find between our main variables of interest. We hypothesized a strong relationship between proportion of residents working in outdoor industries and heat-related health outcomes among those residents during summer heat events, where higher rates of work in industries such as construction, agriculture, and related industries would result in clear increases of heat-related deaths, ED visits, and hospitalizations. We also anticipated a relationship between prevalence of outdoor work and All Internal Cause health outcomes during heat events—given known associations between heat and various bodily systems, as well as the potential for heat-related symptoms to go unrecognized by coroners or medical providers [21]—but that the treatment effect in this case would be more modest. We hypothesized there would be no relationship between prevalence of non-outdoor work and either heat-related or All Internal Cause health outcomes during summer heat events.

In order to examine community-level relationships between heat-related health outcomes and prevalence of outdoor work, frequencies of each outcome were generated by zip code. (Both occupation and health outcomes data are linked to place of residence; our analysis focuses on the type of work performed by community residents rather than the location of that work). Because the outcomes are non-negative count variables, Poisson regression models were used to model the number of events in each zip code for each outcome. Models were adjusted by adding the following covariates into the model: the percent of the population over 65 years of age; percent Hispanic; percent African American; percent Native American; and percent of individuals living below the federal poverty level within the zip code. These variables were considered factors that may also influence heat vulnerability within the zip code. All models were offset by population to convert to a per capita rate. Incident rate ratios and their 95 percent confidence intervals were produced to estimate the increased or decreased rate of morbidity and mortality for each unit increase in outdoor workers. SAS version 9.4 (SAS Institute, Cary, NC, USA) was used for all analyses. *p*-values of less than 0.05 were considered statistically significant.

## 3. Results

### 3.1. Los Angeles Demographic and Geographic Characteristics and Health Outcomes

Table 1 provides overall demographic and geographic characteristics and health outcomes for Los Angeles County based on U.S. Census data. The total population size of the County in 2012 was 9.8 million, and the total civilian workforce aged 16 years and over was 4.5 million. The County covers over 4,000 square miles, an enormous land area that comprises dense urban and coastal communities, as well as more sparsely populated deserts and mountain ranges. Approximately 5.8 percent of the population of the County worked in construction and 0.5 percent worked in agriculture, forestry, fishing and hunting, and mining—a combined 6.3 percent of the total population in these “outdoor” industries. An additional 20.5 percent worked in education services, and healthcare and social assistance. Table 1 also provides statistics on race, age, and individuals living below the federal poverty line.

We identified a total of 246 summer heat event days in Los Angeles County between 2005 and 2010. During these heat events, we found 19 deaths among adults aged 16 years and over that were attributed to heat (0.2 per 100,000 residents), 2011 emergency department (ED) visits attributed to heat (20.5 per 100,000 residents), and 503 hospitalizations attributed to heat (5 per 100,000 residents). In addition, we found 25,131 deaths among adults aged 16 years and over that were attributed to All Internal Causes (256 per 100,000 residents), 263,839 ED visits attributed to All Internal Causes (2687 per 100,000 residents), and 370,546 hospitalizations attributed to All Internal Causes (3774 per 100,000 residents).

### 3.2. Community-Level Variations in Prevalence of Outdoor Work

Table 2 shows the distribution of employment in selected outdoor and non-outdoor industries across Los Angeles County zip codes. The median proportion of residents working in construction per zip code was 5.2 percent, and the median proportion of residents working in agriculture, forestry, fishing and hunting, and mining was 0.3 percent. (The proportion of residents in each of these industries reached as high as 12.9 percent in construction and 12.5 percent in agriculture, forestry, fishing and hunting, and mining). The median proportion of residents working in education services, and healthcare and social assistance, per zip code was 20.9 percent.

### 3.3. Associations between Work and Health Outcomes

Table 3 shows incident rate ratios for each industry sector and various health outcomes of interest, both those attributed to heat and those attributed to All Internal Causes.

In the case of outdoor industries, we found that rates of heat-related ED visits during summer heat events were significantly higher in communities with a greater proportion of residents working in either construction or agriculture, forestry, fishing and hunting, and mining after adjusting for age, race, and poverty. Specifically, each percentage increase in residents working in construction resulted in an 8.1 percent increase in heat-related ED visits during summer heat events, and each percentage increase in residents working in agriculture and related sectors resulted in a 10.9 percent increase in heat-related ED visits. Heat-related hospitalizations were also more frequent during summer heat events in communities with a greater proportion of residents working in construction. Each percentage increase in residents working in this industry resulted in a 7.9 percent increase in heat-related hospitalizations. No significant associations were found between prevalence of work in these outdoor sectors and deaths attributed to heat during summer heat events.

Our analysis found more modest associations between patterns of outdoor work and All Internal Cause health outcomes at the community level after adjusting for age, race, and poverty. Each percentage increase in residents working in construction resulted in a 1.9 percent increase in ED visits and a 1.2 percent increase in deaths during summer heat events. ED visits attributed to All Internal Causes during summer heat events were also higher in communities where more residents work in agriculture, forestry, fishing and hunting, and mining, with each percentage increase in employment in these sectors resulting in a 2.9 percent increase in ED visits. No significant associations were found between prevalence of outdoor work and All Internal Cause hospitalizations.

In the case of non-outdoor industries, we found that heat-related ED visits during summer heat events were significantly higher in communities with a greater proportion of residents working in education services, and healthcare and social assistance, and that both All Internal Cause deaths and ED visits during summer heat events were significantly higher in these communities as well However, the relationships between industry prevalence and these health outcomes were modest. Each percentage increase in residents working in these sectors resulted in a 1.3 percent increase in heat-related ED visits, while the effect on All Internal Cause health outcomes was 0.5 percent or less.

## 4. Discussion

Our analysis of data from Los Angeles County, California, between 2005 and 2010 found strong associations between the proportion of residents working in outdoor industries and rates of heat-related ED visits and hospitalizations during summer heat events. Each percentage increase in residents working in construction resulted in an 8.1 percent increase in heat-related ED visits and a 7.9 percent increase in heat-related hospitalizations during summer heat events, while each percentage increase in residents working in agriculture, forestry, fishing and hunting, and mining resulted in a 10.9 percent increase in heat-related ED visits. The associations we found between prevalence of outdoor work and All Internal Cause deaths and ED visits were modest in effect, but provide further evidence for a relationship between work and community-level health, since many of the cardiovascular, respiratory and renal symptoms associated with All Internal Cause attributions are known to be linked to heat as either acute or chronic manifestations of exposure [33,48,50].

One explanation for these patterns may be increased environmental heat exposure resulting from outdoor work activity; that is, as the proportion of residents working in industries such as construction and agriculture increases, so too does the number of individuals exposed to environmental heat conditions, thereby increasing the risk of heat stroke and heat illness as well as other health complications that may be exacerbated by heat. Other indirect mechanisms may also be at play to shape the heat-health relationship at the community level. Low-wage agricultural workers, for example, may endure housing conditions that increase their heat burden [51]. Additional research is needed on the various mechanisms by which work and community environments shape personal heat health risks, as well as the potential interactions between them [52].

While we found significant associations between certain health outcomes during summer heat events—both heat-related and All Internal Cause—and the proportion of residents working in the “non-outdoor” industries of education services, and healthcare and social assistance, these associations were modest in effect. The strongest association was found between work in these sectors and heat-related ED visits, with each percentage increase in residents working in education services, and healthcare and social assistance resulting in 1.3 percent increase in heat-related ED visits during summer heat events. These results could reflect work-related exposures: some individuals working in these industries may, in fact, be subject to environmental weather conditions in the course of their work—school-based sports coaches, for example, or those working in older buildings without air conditioning—and these effects in turn could manifest in community-level statistics. Alternately, the associations may be driven by confounding variables not included in our model.

Our analysis found no significant associations between proportion of residents working in outdoor industries and heat-related deaths. There are a number of possible explanations for these results. With only 19 deaths attributed to heat during summer heat events among adults aged 16 years and over between 2005 and 2010, there is reason to believe that heat-related deaths may be undercounted in these statewide data, as a number of factors have been shown to contribute to underreporting [9,21,53]. Alternately, the low number of heat-related deaths in our dataset could, in turn, be a consequence of California’s occupational health standard (California Code of Regulations Title 8 Section 3395) designed to prevent heat illness among outdoor workers. An emergency temporary standard went into effect in 2005, and the standard was made permanent the following year [23,54]. The California standard requires employers to provide drinking water, shade structures, and cool-down opportunities for workers when ambient temperatures exceed 80 degrees Fahrenheit. Additional high-heat preventive procedures are required for employers in select industries when temperatures reach or exceed 95 degrees Fahrenheit. Given that the time period of our analysis corresponds to the first five years of implementation of the standard in California, our results may suggest a positive impact of the law in helping to prevent the most extreme consequences of heat exposure on outdoor workers—i.e., heat-related death. Yet, the standard may not be effective enough to eliminate other non-fatal health consequences during summer heat events. Additional research comparing public health data before and after implementation of the standard would help elucidate the impact of the California standard on work conditions and heat-related health outcomes.

The findings from this analysis are subject to several limitations. Specific mortality or morbidity events cannot be linked directly to individuals’ occupations, thereby preventing us from identifying direct mechanisms of exposure in specific industries or work settings. It is also possible that, despite confining our analysis to summer heat events, some of the deaths, ED visits, and hospitalizations in our model—particularly those attributed to All Internal Causes—are not sequelae of environmental heat. Additionally, while air mass data offers a number of advantages over more traditional measures of temperature and humidity, these data do not fully account for the wide range of meteorological variations on the ground, particularly given the many microclimates in Los Angeles County.

These limitations notwithstanding, a notable strength of our analysis is the linking of multiple sources of data to examine the role of work as a potential contributor to community-level health outcomes. By relying on broad community-level health and demographic data, we avoid attribution difficulties inherent in many traditional occupational health studies. The associations we have attempted to draw between work activity and heat-related health outcomes are not dependent on employer records of worker injuries, or on coroners or healthcare providers correctly identifying conditions as work related. What our analysis lacks in specificity at the individual level is therefore compensated for in the aggregate by our ability to identify broad patterns that would otherwise be missed. This is particularly valuable given the absence of data on the incidence of occupational heat-related illness in the U.S. [21].

## 5. Conclusions

This study offers evidence of a relationship between community-level patterns of work and broad public health indicators of heat-related health outcomes, even after other recognized heat-related risk factors such as age and socioeconomic status are taken into account. The results support the notion of work and employment as important social determinants of health and contributors to community-level health inequalities [27,29,55]. In the case of extreme heat events, heat-related morbidity may be clustered predictably and geographically based, in part, on prevalence of residents working in outdoor settings [30]. Replication of this research in other settings, across different time periods, and employing more sensitive measures of industry and occupation would help corroborate the relationship we found between outdoor work and community-level, heat-related health outcomes. Such research efforts would also benefit from improved methods for capturing patients’ fields of work and/or the suspected work-relatedness of health conditions in medical records, thereby enabling more direct connections to be drawn between work and health [56,57].

As the frequency and severity of local heat events are anticipated to increase along with global climate change, our findings point to the need for community-level data on work to be better incorporated into heat-related surveillance and planning efforts. Public health professionals and emergency service providers should consider work and employment as potential risk factors when preparing for and responding to extreme heat events. The results of our analysis also suggest that community-level data on industry and occupation can serve as a valuable tool for anticipating excess burdens on safety net healthcare services during extreme heat events. Additionally, as emergency departments and hospitals respond to heat-related health symptoms that may result from occupational exposures, they would do well to better capture information about patients’ places of work to ensure that the costs of treating these cases are appropriately borne by employers (via workers’ compensation insurance programs) and not shifted to healthcare providers or government programs [58].

Finally, this research points to the need for effective programs and strategies to prevent mortality and morbidity resulting from occupational heat exposure. Employers, unions, community-based organizations, and healthcare providers can serve as resources for training and information about the risks of work in high heat environments and precautions to avoid heat illness [23,24,59]. Voluntary changes to prevailing work practices within industry sectors have also been identified as playing a protective role—rescheduling work shifts to begin during the early morning hours, for example, to limit workers’ activities during the heat of midday [60]. Policies such as California’s heat illness prevention standard can go even further by establishing a legally enforceable framework to effect changes in workplace design and work activities across multiple industries and sectors. Studies have shown that employers often fail to implement common elements of heat illness prevention programs in the absence of such standards [61]. These and other strategies become increasingly necessary as a means to prevent occupational heat-related outcomes and protect the wellbeing of communities at large.

## Figures and Tables

**Table 1:** Population and geography characteristics and health outcomes, Los Angeles County, California.

**Population Characteristics**	
Total population, 2012	9,818,605
Civilian employed population 16 years and over, 2012	4,495,118
Pct. work in construction	5.8
Pct. work in agriculture, forestry, fishing and hunting, and mining	0.5
Pct. work in education services, and healthcare and social assistance	20.5
Pct. population aged 65 and over	11.5
Pct. population identified as Hispanic	48.2
Pct. population identified as African American	8.4
Pct. population identified as Native American	0.5
Pct. individuals living below federal poverty line	17.1
**Geography Characteristics**	
Land area in square miles	4058
Population per square mile, 2012	2419.6
Summer (May–September) heat event days, 2005–2010 ^1^	246
**Health Outcomes**	
Outcomes attributed to heat during summer heat event days, 2005–2010 ^2^	
Total deaths, heat-related	19
Total ED visits, heat-related	2011
Total hospitalizations, heat-related	503
Outcomes attributed to All Internal Causes during summer heat event days, 2005–2010 ^2^	
Total deaths, All Internal Causes	25,131
Total ED visits, All Internal Causes	263,839
Total hospitalizations, All Internal Causes	370,546

^1^ Heat event days defined as days with “oppressive” air masses based on NOAA’s spatial synoptic classification system; ^2^ outcomes limited to individuals aged 16 years and over. NOAA: National Oceanic and Atmospheric Administration.

**Table 2:** Distribution of employment in select industries.

Zip codes in Los Angeles County	276
Pct. work in construction, 2012	
Mean	5.4
Std. deviation	2.7
Median	5.2
Min–Max	0.0–12.9
Pct. work in agriculture, forestry, fishing and hunting, and mining, 2012	
Mean	0.5
Std. deviation	0.9
Median	0.3
Min–Max	0.0–12.5
Pct. work in education services, and healthcare and social assistance, 2012	
Mean	21.2
Std. deviation	6.0
Median	20.9
Min–Max	7.6–64.8

**Table 3:** Incident rate ratios for outcomes attributed to heat during summer heat events, Los Angeles County, 2005–2010.

Industry	Heat Related			All Internal Cause		
	Deaths	ED Visits	Hospital Discharges	Deaths	ED Visits	Hospital Discharges
Construction						
*Unadjusted*	1.000 (0.835, 1.199)	**1.023 (1.005, 1.042)**	1.035 (0.999, 1.072)	**0.958 (0.954, 0.963)**	**1.072 (1.070, 1.073)**	**0.991 (0.990, 0.993)**
*Adjusted* *	1.091 (0.823, 1.446)	**1.081 (1.051, 1.112)**	**1.079 (1.022, 1.138)**	**1.012 (1.004, 1.019)**	**1.019 (1.016, 1.021)**	1.002 (0.999, 1.004)
Agriculture, Forestry, Fishing and Hunting, and Mining						
*Unadjusted*	0.613 (0.190, 1.975)	1.080 (0.988, 1.181)	0.907 (0.740, 1.113)	**0.848 (0.824, 0.873)**	**1.212 (1.205, 1.219)**	**0.977 (0.970, 0.984)**
*Adjusted* *	0.610 (0.151, 2.472)	**1.109 (1.016, 1.211)**	0.884 (0.690, 1.132)	1.019 (0.988, 1.052)	**1.029 (1.019, 1.038)**	1.002 (0.994, 1.011)
Education Services, and Healthcare and Social Assistance						
*Unadjusted*	1.036 (0.948, 1.132)	**1.015 (1.006, 1.025)**	1.008 (0.988, 1.027)	**1.023 (1.020, 1.025)**	**0.984 (0.983, 0.985)**	**1.007 (1.006, 1.008)**
*Adjusted* *	1.033 (0.923, 1.155)	**1.013 (1.001, 1.026)**	1.015 (0.992, 1.039)	**1.005 (1.001, 1.008)**	**1.003 (1.003, 1.004)**	0.999 (0.998, 1.000)

* Adjusted for % over 65 years, % Black, % Hispanic, % Native American, % below poverty level; numbers in bold are statistically significant.
